# Study on Protein Structures of Eight Mung Bean Varieties and Freeze-Thaw Stability of Protein-Stabilized Emulsions

**DOI:** 10.3390/foods11213343

**Published:** 2022-10-24

**Authors:** Hongrui Sun, Jieying Fan, Hongjiao Sun, Guochuan Jiang, Yue Meng, Xianpeng Zeng, Zhiqiang Yang, Xiping Nan, Lining Kang, Xiangying Liu

**Affiliations:** 1 Institute of Agro-Food Technology, Jilin Academy of Agricultural Sciences, Changchun 130033, China; 2 Jilin Jinong Hi-tech Inc., Ltd., Gongzhuling 136100, China; 3 College of Food Science and Engineering, Jilin Agricultural University, Changchun 130118, China

**Keywords:** mung bean protein isolate, freeze-thaw stability, protein structure, albumin and globulin content, emulsion parameters, flexibility measurement, functional properties, fourier-transform infrared spectroscopy, circular dichroism

## Abstract

In order to evaluate the freeze-thaw stability of mung bean protein isolate (MPI)-stabilized emulsions and its relationship with protein structure, proteins of eight mung bean varieties were compared. The results revealed that MPIs prepared from all eight varieties were mainly composed of five subunit bands, with albumin and globulin content ranges of 188.4–310.3 and 301.1–492.7 mg/g total protein, respectively. Protein structural analysis revealed that random coil structure (32.34–33.51%) accounted for greater than 30% of MPI secondary structure. Meanwhile, analysis of protein properties revealed emulsifying activity index (EAI), emulsifying stability index (ESI) and flexibility value ranges of 6.735–8.598 m^2^/g, 20.13–34.25% and 0.125–0.182, respectively. Measurements of freeze-thaw stability of MPI emulsions demonstrated that exposures of emulsions to multiple freeze-thaw cycles resulted in significantly different emulsion creaming index, oiling-off, particle size and zeta potential values for the various emulsions. Moreover, the stabilities of all eight protein emulsions decreased with each freeze-thaw cycle, as demonstrated using optical micrographs. The correlation analysis method was used to study the correlation between the original structures, emulsifying properties of proteins and the freeze-thaw stability of MPI emulsions. Correlation analysis results revealed significant relationships between albumin content, subunit bands with a molecular weight of 26.9 kDa and emulsifying properties were significantly related to the freeze-thaw stability of MPI emulsion. Thus, by determining these indicator values, we can predict the freeze-thaw stability of MPI-stabilized emulsions.

## 1. Introduction

An emulsion is a type of dispersion system consisting of one or more liquids dispersed as droplets in another immiscible liquid. Due to differences in density and polarity between emulsion liquids, unstable emulsions require additions of emulsifiers to maintain system stability [1]. Plant proteins, which are generally amphiphilic and can serve as emulsifiers when added to emulsions, can spontaneously form stable interfacial films at the two-phase interface that reduce interfacial tension and reduce mutual aggregation between liquids to effectively improve emulsion stability [2]. At the same time, most plant proteins are digestible and highly nutritious, thus aligning with current healthy lifestyle recommendations. Nevertheless, even though plant proteins are excellent natural emulsifiers, their ability to stabilise emulsions is readily undermined by changes in system pH, ionic strength and temperature [3]. For example, some emulsions are incorporated into foods that must be frozen to extend shelf life (such as sausages and beverages) or to provide desired food characteristics (e.g., ice cream and quick-frozen food). However, protein emulsions become unstable after multiple freeze-thaw cycles, resulting in emulsion instability leading to flocculation and generation of floating fat globules, which seriously decrease food quality. Freeze-thaw instability of protein emulsions is mainly caused by the crystallisation of water/oil phases and changes in protein structure and droplet microenvironmental characteristics [4,5]. So far, numerous techniques have been proposed to improve the freeze-thaw stability of protein emulsions based on strategies for reducing ice crystal growth, improving product composition and shear conditions, enhancing cryoprotection (adding sugars and polyols, etc.), or strengthening interface integrity.

At present, research on the freeze-thaw stability of plant protein-stabilized emulsions has mainly been focused on soy protein isolate (SPI) [1,2,5]. SPI, which is widely used in the food industry, has a protein content of greater than 90%, high nutritional value and good functional properties [1,5]. However, studies have shown that soy protein used in foods consumed by infants and young children is associated with health risks. For example, Ogawa et al. (2000) tested blood serum samples of patients with soy allergies and detected three main soy protein allergens, Gly m Bd 30K, Gly m Bd 28K and Gly m Bd 60K, of which the allergen with the greatest sensitising activity was Gly m Bd 30K, a component of soybean 7S globulin [6]. Due to health risks associated with the use of soybean proteins in food, other natural emulsifiers are currently being sought for use in stabilizing emulsified foods stored in frozen environments. MPI holds promise as a soybean protein substitute, as demonstrated by Brishti et al. (2020), who found it to possess superior amino acid composition (high lysine, isoleucine and cystine content) and functional properties (such as gelling and foaming capacity) as compared with soybean protein [7]. In addition, MPI has been shown to have low allergenicity and beneficial health effects for improving lipid metabolism and preventing non-alcoholic fatty liver. Due to these characteristics, MPI has been recommended for use as a supplement in infant formula as a high-quality plant-based protein source [8,9,10]. Currently, MPI is generated in large quantities as a by-product of the mung bean starch production process but is greatly underutilised as a protein resource [11]. Therefore, to avoid wasting this important resource, it is necessary to find ways to better utilise MPI as a food ingredient.

The use of proteins as food additives to improve food quality is a common practice in the food industry. It has been reported that low temperatures can trigger protein denaturation and aggregation and alter protein functional properties. In addition, shear, extrusion, concentration and other effects during freeze-thaw cycles can alter protein structure, intermolecular forces and physicochemical properties [2]. Notably, the physicochemical and functional properties of proteins are closely dependent on their structural properties [12]. Thus, improving the freeze-thaw stability of protein emulsions and clarifying the relationship between protein structure and freeze-thaw stability of protein emulsions are prerequisites for the development of antifreeze proteins for use in frozen environments. To date, efforts by researchers to improve the freeze-thaw stability of protein-stabilized emulsions have mainly focused on protein structural modification strategies utilising various physical and chemical interventions. However, an extensive literature search that we conducted revealed no reported studies of MPI emulsion freeze-thaw stability or its relationship with protein structure, prompting this study. Emulsification ability is an important property of plant protein-based functional food ingredients [13]. Based on this, this study selected eight mung bean varieties with large differences in structure and emulsification properties. Here we comparatively analysed differences in subunit composition, protein composition, emulsifying properties, the flexibility of proteins extracted from eight mung bean varieties and freeze-thaw stability of protein-stabilized emulsions. Our results were analysed to clarify the relationship between MPI structure and freeze-thaw stability of MPI-stabilized emulsions. Ultimately, these results should motivate crop breeders and growers to develop and cultivate mung bean varieties that contain proteins with special properties. Extending the applicability of MPI to frozen foods will stimulate new MPI research initiatives to uncover other important discoveries with scientific and practical significance that may lead to greater utilisation of mung beans as a food protein resource.

## 2. Materials and Methods

### 2.1. Materials

Mung bean varieties (MB1-MB8) were collected from eight regions of China by the Institute of Crop Resources of Jilin Academy of Agricultural Sciences (Jilin, China). Their protein contents were 23.90%, 24.98%, 25.33%, 25.12%, 24.72%, 24.26%, 23.80% and 24.30%, respectively. Soybean oil was purchased from the Shandong Luhua Group (Yantai, China). Trypsin was purchased from Sigma (250 U/mg, St. Louis, MO, USA). Sudan III and Coomassie Brilliant Blue G-250 were purchased from Shanghai Yuanye Biotechnology Co., Ltd. (Shanghai, China). Disodium hydrogen phosphate and sodium dihydrogen phosphate were purchased from the Tianjin Guangfu Institute of Fine Chemicals (Tianjin, China).

### 2.2. Preparation of MPIs

Mung beans were mixed with distilled water in a ratio of 1:10 and soaked at 4 °C for 12 h. To further process the soaked mung beans, they were first pulverised then the pH of the pulverised bean suspension was adjusted to 9.0 with NaOH (2M). Thereafter, the beans were stirred at low speed for 30 min in a water bath at 40 °C. Next, the bean-water mixture was centrifuged (4000× *g*) for 20 min. The supernatant was saved, and its pH was adjusted to 4.5 with HCl (2 M), then the mixture was allowed to stand overnight at 4 °C. Next, the mixture of liquid and precipitate was centrifuged (4000× *g*) for 5 min, and the supernatant was discarded. The pellet was washed with water, centrifuged and washed again twice then it was resuspended in distilled water. After the pH was adjusted to 7.0, each MPI was freeze-dried [14].

### 2.3. Determination of Albumin and Globulin Content

The test method of Du et al. (2017) was used to extract mung bean albumin and globulin protein, with slight modifications [14]. Mung beans were crushed using a pulveriser and passed through a 60-mesh sieve, then the crude fat in the resulting mung bean powder was removed via the Soxhlet extractor method. First, defatted mung bean powder was mixed with distilled water in a ratio of 1:10 (by weight), placed in a 40 °C water bath, then stirred at low speed for 30 min. Next, the mixture was centrifuged (4000× *g*, 20 min, 4 °C), then the supernatant was filtered, and the filtrate was retained. Thereafter, 500 mL of distilled water was added to the pellet, and the extraction was repeated using the same procedure as used for the first extraction mentioned above. Supernatants obtained from the two extractions were combined and freeze-dried to yield albumin. Albumin content was determined using an automatic Kjeldahl nitrogen analyser.

The precipitate remaining after albumin extraction was suspended in 0.5 M NaCl, then the tube containing the suspension was placed in a 40 °C water bath and shaken at low speed for 30 min. The suspension was then centrifuged (4000× *g*, 20 min, 4 °C), and the supernatant was collected and filtered. Next, 500 mL of NaCl (0.5 M) was added to the pellet followed by re-extraction of the pellet using the same extraction procedure as used before. Supernatants obtained from both extractions were combined and freeze-dried to yield globulin. Globulin content was determined using an automatic Kjeldahl nitrogen analyser.

### 2.4. Fourier-Transform Infrared Spectroscopy (FT-IR)

To an appropriate amount of MPI sample, a certain amount of KBr was added, and then the solid mixture was ground into powder, pressed into thin slices, and analysed using a Fourier-transform infrared (FT-IR) spectrometer (Nicolet iS50, Thermo Fisher, Waltham State, MA, USA). A broad scanning range was used for detection (4000–400 cm^−1^), as previously described [1].

### 2.5. Circular Dichroism (CD)

MPI was dissolved in a phosphate buffer solution (0.1 M, pH 7.0), then the protein concentration was adjusted to 100 μg/mL. A circular dichroism spectrometer (ChirascanqCD, Applied Photophysics Ltd., Leatherhead, UK) was used to scan the sample in the far ultraviolet range (260–190 nm) using a scanning bandwidth of 1.0 nm. Proportions of α-helix, β-sheet, β-turn and the random coil of overall protein secondary structure were calculated using CDPro software [14].

### 2.6. SDS-PAGE

Sodium dodecyl sulphate-polyacrylamide gel electrophoresis (SDS-PAGE) was conducted using the method of Brishti et al. (2020) with a minor modification [7]. First, MPI (0.5 mg/mL) was dissolved in 0.1 M NaOH solution. Next, 10 μL of the protein solution was mixed with 10 μL of loading buffer and heated in a boiling water bath for 5 min (to thermally denature the protein) followed by rapid cooling. Denatured protein preparations (10 μL/lane) were next loaded into wells of a polyacrylamide gel (5% stacking gel, 12% separating gel). Electrophoresis was conducted using a voltage of 80 V until the dye band reached the boundary between the stacking and separating gel layers. The voltage was then increased to 120 V, and electrophoresis was allowed to continue until the dye band reached the bottom of the separating gel. The gel containing separated MPI was stained with Coomassie Brilliant Blue G250, then destained using standard procedures.

### 2.7. Emulsification Determination

The degree of protein emulsification was determined using the method of Feng et al. (2020) with minor modifications [15]. First, the MPI sample was dissolved in phosphate buffer solution (0.2 M, pH 7.0), then the protein concentration was adjusted to 0.4% (*m/v*). The protein solution was next mixed with soybean oil in a ratio of 3:1 (*v/v*) for 1 min using a high-speed blender (Ultraturrax T-25, IKA Labortechnik, Staufen, Germany) (10,000 r/min). Next, 100 μL was removed from the bottom of the mixture and added to 10 mL of 0.1% SDS solution and mixed well. Absorbances were measured at 0 min and 10 min after homogenisation using the following formula:

(1) $$EAI\left(\frac{m^{2}}{g}\right)=2\times 2.303\times A_{0}\times \frac{N}{C\times \left(1-\emptyset \right)\times 10^{4}}$$

(2) $$ESI\left(\%\right)=\frac{A_{10}}{A_{0}}\times 100$$

where *N* represents the dilution factor, *C* represents the protein concentration in the protein solution before the emulsion was formed (g/mL), ∅ represents the volume fraction of oil in the emulsion (*v*/*v*), *A*_0_ represents the absorbance measured at 0 min, and *A*_10_ represents the absorbance measured at 10 min; *A*_0_ and *A*_10_ are used to calculate the emulsifying activity index (*EAI*, m^2^/g) and emulsifying stability index (*ESI*, %).

### 2.8. Flexibility Measurement

Based on the method of Kato et al. (1990) with a slight modification, MPI sensitivity to trypsinisation was measured to determine protein flexibility [16]. First, 250 μL trypsin solution (Tris-HCl buffer: 0.05 mol/L, pH 8.0; trypsin concentration of 1 mg/mL) and 4 mL of MPI solution (protein concentration 1 mg/mL; volume ratio of protein solution: trypsin solution of 16:1) were mixed well and then placed in a constant temperature water bath (38 °C). Trypsinisation was allowed to proceed for 5 min, then trichloroacetic acid was added (5%, 4 mL) to stop the reaction. Next, the tube was centrifuged (6000× *g*, 10 min), and the supernatant was removed. Next, the absorbance of the supernatant was measured at a wavelength of 280 nm as an indicator of protein flexibility.

### 2.9. Emulsion Preparation

MPI emulsion was prepared based on the test method of Wang et al. (2020) with a slight modification [1]. First, MPI was dissolved in phosphate-buffered saline (0.01 M, pH 7.0) and stirred with a magnetic stirrer for 30 min. Next, the protein concentration was adjusted to 3%, then the solution was placed in a refrigerator at 4 °C for 12 h to allow hydration of the protein. Soybean oil was added to the protein solution to a final concentration of 5% (*v/v*) oil, then the mixture was homogenised for 5 min (10,000 r/min) to form an emulsion.

### 2.10. Freeze-Thaw Cycle

In order to test the freeze-thaw stability of MPI emulsion, the freshly prepared emulsion was transferred to a 10-mL plastic tube then the tube was sealed and stored in a freezer at −18 °C for 24 h. Next, the frozen emulsion was thawed at 20 °C for 2 h, and its freeze-thaw stability was assessed. This procedure was repeated for a total of three freeze-thaw cycles [2].

### 2.11. Creaming Index (CI) Determination

To determine the MPI emulsion creaming index (*CI*), the freshly prepared MPI emulsion was placed into a 10-mL emulsification tube. The tube was stored at −18 °C for 24 h then the frozen emulsion was thawed, and layering (stratification) of the emulsion was noted. Generally, after emulsion stratification occurs, the upper layer is referred to as the emulsified layer and the lower layer is referred to as the serum layer [2]. The creaming index (*CI*) was calculated using the following formula:

(3) $$CI\left(\%\right)=\frac{H_{S}}{H_{E}}\times 100$$

where *H_E_* represents the total height of the emulsion (cm), and *H_S_* represents the height of the serum layer (cm).

### 2.12. Oiling-Off Determination

For oiling-off determinations, 16 g of MPI emulsion and 4 g of dye solution (0.015 g Sudan III and 1000 mL soybean oil mixed thoroughly), were combined and then thoroughly mixed. Next, the mixture was centrifuged at 16,000× *g* for 20 min then the supernatant was collected, and its absorbance was measured at a wavelength of 508 nm. Soybean oil served as the control [2]. The formula used to calculate oil yield was as follows:

(4) $$\varphi (\%)=\frac{m_{0}\times (a-1)}{m_{e}\times \varphi_{d}}\times 100$$

where *φ_d_* represents the mass fraction of the oil phase within the MPI emulsion, *m_0_* represents the mass of dye solution (g), *m_e_* represents the mass of emulsion (g), and *a* represents the ratio between pre- and post-centrifugation absorbances of dyed emulsion (g).

### 2.13. Optical Microscopy

To determine freeze-thaw stability, the MPI emulsion microstructure was analysed using optical microscopy. Before and after each freeze-thaw cycle, the emulsion was vortexed then a small amount was transferred to the center of a microscope slide, covered with a coverslip, then viewed and imaged under a microscope at 100× magnification [1,2].

### 2.14. Determination of Emulsion Particle Size and Zeta Potential

The protein emulsion was diluted 10-fold with phosphate-buffered saline (0.01 M, pH 7.0) then particle size distribution, average particle size and zeta potential values of the emulsion were determined using a nanoparticle size potentiometer (Zetasizer Nano ZS, Malvern, UK). For parameter settings, the protein particle refractive index was set to 1.460 and the dispersant refractive index was set to 1.330 [1,17].

### 2.15. Data Analysis

All analyses were performed in triplicate. Data are expressed as the mean ± Standard Deviation (SD). Significant differences (*p* < 0.05) in results obtained between different indicators were analysed via correlation analysis that was conducted using statistical SPSS software version 16 (SPSS Inc., Chicago, IL, USA). Figures were drawn using Origin software Version 8.5 (OriginLab, Northampton, MA, USA).

## 3. Results and Discussion

### 3.1. Analysis of Structural and Emulsifying Properties of Mung Bean Proteins of Eight Mung Bean Varieties

#### 3.1.1. Protein Subunit and Composition Analysis

Compositional differences have been shown to influence mung bean storage protein properties, including nutritional quality, hydration properties, surface properties and protein interactions, among others [18]. MPI is rich in storage protein components that mainly include globulin, albumin, gliadin and glutelin, with high contents of globulin and albumin reported [14,19]. In this study, proteins of eight mung bean varieties were separated using the Osborne method, yielding two protein components with high content levels, namely albumin and globulin. As shown in Table 1, albumin and globulin contents of mung bean storage proteins obtained from the eight mung bean varieties tested here differed significantly. Albumin contents ranged from 188.4–310.3 mg/g of total protein, with an average albumen content of 253.8 mg/g of total protein. MPI_4_ had the highest albumin content (310.3 mg/g of total protein), and MPI_8_ had the lowest albumin content (188.4 mg/g of total protein). As compared with albumin content values, globulin content values determined for MPIs of the eight mung bean varieties were relatively higher, ranging from 301.1–492.7 mg/g of total protein, with an average globulin content of 366.5 mg/g of total protein. MPI_1_ and MPI_5_ possessed higher globulin contents of 492.7 and 483.4 mg/g of total protein, respectively, while MPI_6_ possessed significantly lowest globulin content (301.1 mg/g of total protein), which was significantly different from other varieties of MPI. It has been reported that mung bean albumin has good solubility and contains high amounts of various essential amino acids, highlighting its value as a high-quality protein with high nutritional value as compared to other MPI components [14]. Among the many factors that affect protein emulsifying and interface properties, protein solubility is a key factor required for molecular stabilisation of the oil-water interface [14,20]. Notably, proteins with good solubility in water can readily dissolve in aqueous solutions and possess increased molecular mobility for engaging in rapid adsorption to the oil-water interface, thereby increasing emulsification [1,2,21].

**Figure 1:**
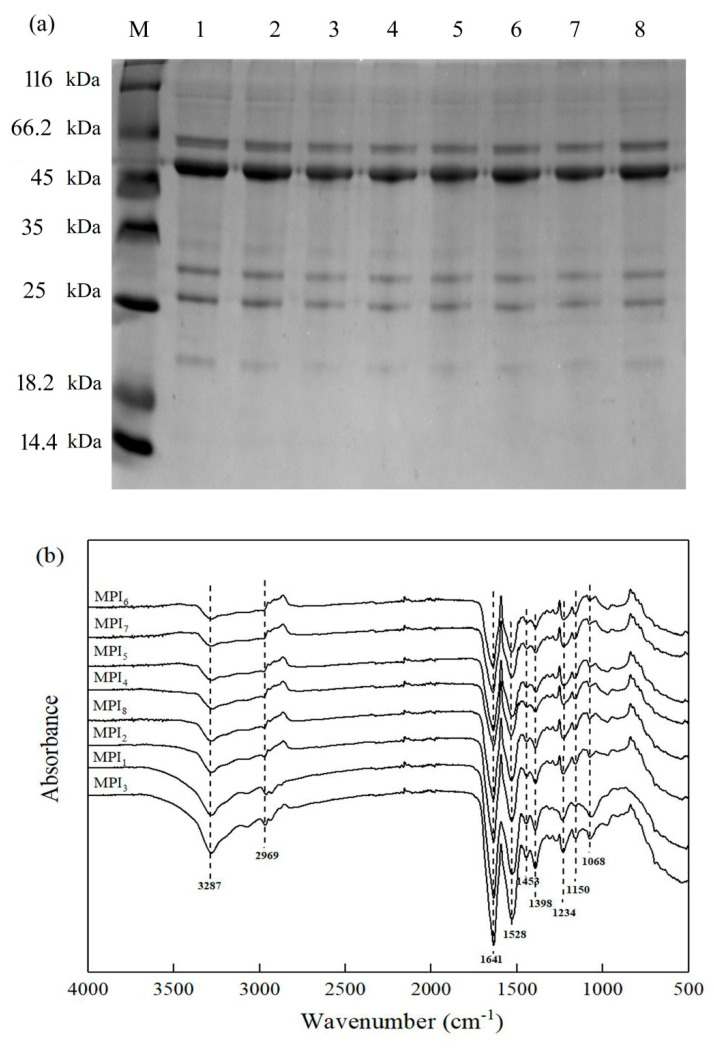
Structure analysis of proteins from eight mung bean varieties: (**a**) SDS-PAGE showingprotein bands of MPI preparations from eight mung bean varieties; M represents the protein size standard; proteins in lanes 1–8 are proteins obtained from eight mung bean varieties (corresponding to MPI_1_–MPI_8_, respectively). (**b**) FT-IR spectra of proteins from eight mung bean varieties.

As a “key” to unlocking structure-activity relationships of proteins, protein subunit composition can be used to effectively explain the many functional properties of proteins. Indeed, numerous studies have shown that protein subunit compositions and different ratios between subunits can affect food processing performance and final food product quality [7]. Here, the results of SDS-PAGE electrophoresis (Figure 1a, Table 1) demonstrated that MPI samples in lanes 1–8 each comprised 5 bands with molecular weights of 21.4, 26.9, 31.4, 52.9 and 63.1 kDa, and their relative content ranges were determined to be 3.70–8.61, 12.89–15.09, 13.02–15.31, 36.69–45.35 and 22.55–26.18%, respectively. Among them, the band with the greatest intensity is found in the size of 52.9 kDa. These results aligned with results reported by Brishti et al. (2020) describing five main MPI bands on SDS-PAGE gels with molecular weights of 15, 25, 26, 50 and 65 kDa [7]. Previously, Mendoza et al. (2001) reported similar results for MPI 7S globulin subunit sizes (16 kDa, 28 kDa), 11S globulin subunit sizes (24, 40 kDa) and 8S globulin subunit sizes (26, 32, 48 and 60 kDa) [22].

#### 3.1.2. Secondary Structures of Proteins from Eight Mung Bean Varieties

Here, Fourier-transform infrared (FT-IR) and circular dichroism (CD) spectral analyses were conducted to obtain secondary structure information for mung bean proteins of eight mung bean varieties. FT-IR spectroscopy is an important method for analysing protein secondary structure that can provide useful protein composition information [14]. As shown in Figure 1b, FT-IR absorption spectra of proteins from the eight mung bean varieties were similar, with O-H and N-H stretching vibration peaks appearing near 3287 cm^−1^ and 2969 cm^−1^. Meanwhile, stretching vibration peaks appearing near 1641 cm^−1^, 1528 cm^−1^, and 1398 cm^−1^ corresponded to amide I, amide II and amide III bonds, respectively. With regard to FT-IT spectra obtained for MPIs by other researchers, Du et al. (2017) observed characteristic FT-IR spectral absorption peaks near 1688 cm^−1^, 1515 cm^−1^ and 1300 cm^−1^, aligning with the results of this study [14]. In another study, Brishti et al. (2021) prepared MPI using three drying techniques: freeze-drying, spray-drying and air-drying, then compared the FT-IR spectra of the three protein samples. Characteristic peaks that were detected for the three protein samples were consistent with peaks corresponding to known secondary structures of legume proteins, namely 1700–1600 cm^−^^1^ (amide I bond, C=O stretching vibration), 1580–1500 cm^−^^1^ (amide II bond, intramolecular and intermolecular C-N stretching vibration and N-H bending) and 1400–1200 cm^−^^1^ (amide III bond, C-N stretching vibration) [23]. Importantly, the absorption peak of the amide I bond, which corresponds to the C=O stretching vibration, is most useful for assessing the secondary structure of a protein. When the secondary structure of the protein was dominated by the α-helix structure, the absorption peak of the C=O stretching vibration occurred in the region of 1650–1658 cm^−1^. When the β-sheet structure was dominant, C=O stretching vibration appeared at an absorption vibration peak around 1610–1640 cm^−1^ [14,24]. Notably, stretching vibration peaks observed for MPIs of all eight mung bean varieties were found within the spectral region around 1641 cm^−1^, indicating that the ordered structure of mung bean protein was predominantly based on the β-sheet structure.

In this work, a CD spectrometer was used to scan MPI solutions in the far ultraviolet range (190–260 nm) to obtain compositional information associated with MPI secondary structures. As shown in Table 1, α-helix, β-sheet, β-turn and random coil content ranges as determined for the eight mung bean varieties were 17.76–21.72, 28.15–32.86, 16.35–16.98 and 32.34–33.51%, respectively, and the average values were 19.33, 30.96, 16.65 and 33.05%, respectively. Of the main forms of protein secondary structure (α-helix, β-sheet, β-turn, random coil), α-helix and β-sheet structures greatly influence spatial distributions of hydrophilic and hydrophobic amino acids that can greatly influence protein amphiphilicity that, in turn, affect protein surface activity [17]. Generally, protein molecules with high α-helix content tend to form compact, cavity-free, conformationally stable structures that are not highly flexible [25]. Secondary structures of proteins from the eight mung bean varieties contained the highest proportions of random coil and β-sheet, followed by α-helix and β-turn. Among these four structural conformations, random coil and β-sheet accounted for more than 60% of the overall secondary structure of MPI, indicating that MPI possesses a low degree of order, which is crucial for improving protein functional properties [26,27]. The contents of α-helix and β-sheet of proteins from the eight mung bean varieties varied greatly, and the differences were significant (Table 1). Meanwhile, the β-sheet contents were higher than the α-helix contents, which was consistent with the results of FT-IR (Figure 1b). 

#### 3.1.3. Emulsification Characteristics of Proteins from Eight Mung Bean Varieties

MPI has an amphiphilic structure, due to its amino acid composition, which includes both hydrophobic and hydrophilic residues. This configuration allows the protein to quickly adsorb to the oil-water interface, reduces interfacial tension, and enables the protein to form a protective film. Protein emulsifying properties include emulsifying activity and emulsifying stability. Emulsifying activity is defined as the ability of a protein to participate in emulsion formation, while emulsifying stability refers to the ability of a protein to maintain emulsion droplets in a uniform distribution without flocculation, coalescence and fat globule floatation during a specified period of time [15,28]. As shown in Table 1, the EAI and ESI of MPI obtained from the eight mung bean varieties tested here differed significantly. EAI and ESI ranges as determined for the eight mung bean varieties were 6.735–8.598 m^2^/g and 20.13–34.25%, respectively. Among them, MPI_4_ had the highest EAI and MPI_1_ had the highest ESI. Meanwhile, albumin contents of MPI_1_ and MPI_4_ were 309.2 and 310.3 mg/g of total protein, respectively, which were significantly higher than other experimental groups. This indicates that the albumin content in MPI has a certain correlation with its EAI and ESI.

The emulsifying ability of a protein mainly depends on its internal molecular structure and its physical and chemical properties [12,15]. Among the many factors that affect emulsifying properties of proteins, molecular flexibility is a decisive factor [20]. The molecular flexibility of a protein is the embodiment of intra- or intermolecular interaction forces among molecules. A protein molecule with good molecular flexibility can quickly change its conformation when the external environment changes [29]. Protein flexibility has attracted increasing attention due to its key role in determining protein functional properties, especially interface functional properties. As shown in Table 1, flexibility values differed significantly among proteins from eight mung bean varieties, with a rank order of values of MPI_4_ > MPI_1_ > MPI_2_ > MPI_5_ > MPI_3_ > MPI_8_ > MPI_6_ > MPI_7_. Notably, similar rankings were obtained for EAI and ESI. Collectively, the results revealed that emulsifying activity, emulsifying stability and flexibility values of MPI_1_ and MPI_4_ were significantly higher than the corresponding values obtained for the other six mung bean proteins. With regard to the emulsion formation process, protein molecules with high molecular flexibility can form more stable oil-water interface films and thus possess better emulsifying activities and emulsifying stabilities than protein molecules with low flexibility [30].

### 3.2. Freeze-Thaw Stability of MPI Emulsions

#### 3.2.1. Effects of Freeze-Thaw Cycles on Particle Size and Zeta Potential of MPI Emulsions 

**Figure 2:**
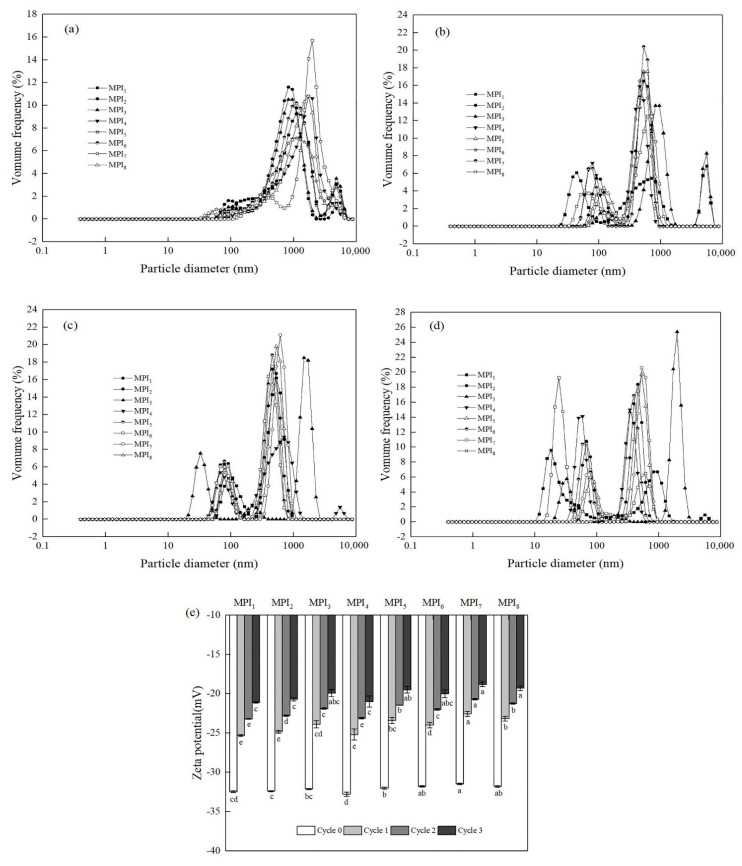
Effect of freeze-thaw cycle number on particle size distribution and zeta potential of MPI emulsions: (**a**) Particle size distribution of MPI emulsions without freeze-thaw treatment; (**b**–**d**) depict particle size distributions of MPI emulsions after 1, 2 and 3 freeze-thaw cycles, respectively; (**e**) Effect of freeze-thaw cycle number on zeta potential of MPI emulsions; a–e values labelled for each freeze-thaw cycle using different letters indicate significant differences (*p* < 0.05).

Freeze-thaw treatment of a protein emulsion is the most destructive and definitive test of emulsion stability [31]. In order to intuitively understand the effect of multiple freeze-thaw cycles on the freeze-thaw stabilities of MPI emulsions, we measured the mean particle sizes and zeta potentials of emulsions. Table 2 and Figure 2 respectively depict the effects of freeze-thaw cycles on mean particle size and particle volume distributions within MPI emulsions. After three freeze-thaw cycles, particle size distributions of MPI emulsions changed from an original unimodal or bimodal distribution to a multimodal distribution. Moreover, the mean particle size of the protein emulsion increased from 343.4–451.7 nm to 722.9–1997.0 nm, thus indicating that the mean particle size increased significantly as emulsion stability deteriorated. In a related recent study, Feng et al. (2020) studied the effect of freeze-thaw cycles on the stability of peanut protein emulsion and found that the emulsion mean particle size increased from 5.61 nm to 32.7 nm after three freeze-thaw cycles; after five freeze-thaw cycles, emulsion particle size peaked at 91.3 nm [15]. In another study conducted by Zang et al. (2018), after three freeze-thaw cycles, the mean particle size of the protein emulsion was 5.53 times that of the original emulsion and the particle size distribution changed from a unimodal distribution to a multimodal distribution [32]. Additionally, other studies have shown that a freeze-thaw cycle destroys the relatively stable state of an emulsion, leading to flocculation and droplet coalescence. These results aligned with earlier studies conducted by Noshad (2015) and Palazolo (2011) using emulsions of SPI and oil, which revealed that after exposure of the emulsion to one freeze-thaw cycle, emulsion mean particle size increased and particle size distributions changed from a single peak to a triple peak, as observed here [2,33]. Mechanistically, an increase in emulsion particle size after multiple freeze-thaw cycles has been attributed to the generation of ice crystals during freezing and thawing that rupture the interfacial film and promote subsequent droplet aggregation [34]. Meanwhile, results reported by Zhao et al. (2016) demonstrated that soy protein emulsion particle size increases that occur during freeze-thawing result from changes in non-covalent interactions (hydrogen bonds, hydrophobic interactions, etc.), which act as direct driving factors of protein aggregate formation [35].

Zeta potential is an important indicator used to characterise the stability of colloidal dispersions [32]. Here, zeta potential value (Figure 2e) ranges of MPI-stabilized emulsions of eight mung bean varieties after 0 to 3 freeze-thaw cycles were −32.78 to −31.46, −25.32 to −22.56, −23.21 to −20.68, and −21.1 to −18.8, respectively. Compared with the original MPI emulsion (cycle 0), absolute values of emulsion zeta potential values decreased with each additional freeze-thaw cycle treatment. Among them, zeta potential value ranges of MPI_1_-stabilized emulsion after 0 to 3 freeze-thaw cycles were −32.45, −25.32, −23.21, and −21.10, respectively. Zeta potential value ranges of MPI_4_-stabilized emulsion after 0 to 3 freeze-thaw cycles were 32.78, −25.20, −23.10, and −21.00, respectively. Under the same conditions, the absolute values of zeta potential values of MPI_1_- and MPI_4_-stabilized emulsions were significantly higher than those of other MPI-stabilized emulsions. Mechanistically, as the absolute zeta potential value of an emulsion increases, repulsive forces between particles increase, leading to increased emulsion system stability. Consequently, emulsions with absolute zeta potential values exceeding 30 mV are stable, due to sizeable electrostatic repulsion forces between droplets [1,36]. Additionally, zeta potential data obtained in other studies suggest that the stability of emulsions decreases after freeze-thaw cycles, aligning with emulsion particle size data. For example, Wang et al. (2020) conducted freeze-thaw cycle experiments on a soybean protein emulsion and found that the absolute value of the emulsion zeta potential value gradually decreased as the number of freeze-thaw cycles increased. Here, zeta potential values of the protein emulsion after 0 to 3 freeze-thaw cycles were −26.5, −24.0, −22.0 and −17.5, respectively, indicating increasing emulsion instability as freeze-thaw cycle number increased [1], as demonstrated by Zang et al. (2018) [32]. Taken together, results of numerous studies have demonstrated that the absolute value of protein emulsion zeta potential value decreases with increasing freeze-thaw cycle number, indicating that freeze-thawing causes emulsion destabilisation and deterioration. Mechanistically, decreased emulsion zeta potential after multiple freeze-thaw cycles can be attributed to ice crystal formation that enhances the ionic strength of liquid in nonfrozen areas around emulsion droplets and thus may block electrostatic repulsion between charged droplets. When the emulsion freezes, ice crystals form that penetrate the oil droplets, destroying the interfacial film at the oil-water interface and thus facilitating emulsion coalescence after thawing [32,37].

#### 3.2.2. Effects of Freeze-Thaw Cycle Number on Creaming Index and Oiling-Off of MPI Emulsions

After an emulsion passes through multiple freeze-thaw cycles, interactions between droplets change, such that the gravitational forces acting on droplets can exceed droplet brownian motion effects that maintain droplets in suspension. As a result, fat globules float upwards, due to the difference in density between the fat globules and the continuous phase, resulting in creaming or sedimentation. Ultimately, this process, which is based on the instability of fat globules after freeze-thaw-induced conversion of emulsified fat into free fat, can be monitored by calculating the degree of coalescence based on the free fat content of the emulsion [1]. Therefore, creaming index and oiling-off values are important indicators of emulsion stability, with reductions of both of these values corresponding to improved protein emulsion freeze-thaw stability [32].

As shown in Table 2 and Figure S1, stable protein emulsions generated from proteins of eight mung bean varieties all exhibited varying degrees of creaming and oiling-off after undergoing multiple freeze-thaw cycles. Nevertheless, the creaming index and oiling-off values of all emulsions consistently trended upward as an indication of increasing emulsion instability as the freeze-thaw cycle number increased. Notably, these results aligned with results reported by Palazolo et al. (2011) indicating that severe detrimental effects resulted from the freeze-thawing of an emulsion composed of a mixture of SPI and oil phases. These effects included severe creaming and oiling effects and signs of severe emulsion instability, including the development of a top layer of free oil accompanied by a middle layer of coagulated emulsion and a bottom turbid aqueous layer [2]. Furthermore, Yu et al. (2018) reported that as the number of freeze-thaw cycles increases, the tendency of an SPI emulsion to undergo oiling-off increases; after the first, second and third freeze-thaw exposures, SPI emulsion oiling-off values were 4%, 6%, and 18.63%, respectively [38]. Similar results were obtained by Zang et al. (2018), who showed that with increasing freeze-thaw cycle number, creaming index and oiling-off values of the protein emulsion increased, emulsion stability gradually decreased, and separation of oil and water phases occurred [32]. In this work, similar MPI emulsion changes were observed after multiple freeze-thaw cycles. As shown in Table 2, CI value ranges of MPI-stabilized emulsions of eight mung bean varieties after 1 to 3 freeze-thaw cycles were 10.00–57.14%, 12.00–60.00%, and 12.87–69.11%, respectively, with an average of 29.90%, 34.10%, and 38.50% respectively. The oiling-off value ranges were 14.12–26.53%, 15.32–26.94%, and 16.10–31.09% respectively, and the average values were 19.90%, 21.40%, and 22.80% respectively. Specifically, CI and oiling-off values obtained after multiple freeze-thaw cycles differed significantly among the eight MPI emulsions tested here, with rank order results for oiling-off of MPI_4_ < MPI_1_ < MPI_2_ < MPI_6_ < MPI_3_ < MPI_5_ < MPI_8_ < MPI_7_ and rank order results for CI of MPI_4_ < MPI_1_ < MPI_6_ < MPI_2_ < MPI_3_ < MPI_5_ < MPI_8_ < MPI_7_. Importantly, the creaming phenomenon reflects emulsion particle size; generally, a smaller emulsion particle size is associated with a lower creaming index value, with the rank order of CI values confirming the accuracy of the particle size data. Notably, the emulsion stabilized by MPI_4_ had the lowest CI and oiling-off values after multiple freeze-thaw cycles. Based on the albumin content (Table 1), EAI (Table 1), ESI (Table 1), and flexibility (Table 1) of proteins, along with the D_4,3_ (Table 2 and Figure 2a–d), CI (Table 2 and Figure S1), zeta potential (Figure 2e) and oiling-off values (Table 2) of MPI-stabilized emulsions of eight mung bean varieties, it can be observed that the freeze-thaw stability of MPI-stabilized emulsion was related to the albumin content, EAI, ESI, and flexibility values of MPI. Zang et al. (2018) showed that the instability of emulsion after multiple freeze-thaw cycles appears to be due to several factors. First, when ice crystals form in an emulsion, oil droplets in unfrozen areas near ice crystals become more tightly packed together, thus destroying the interface film and promoting droplet coalescence. Secondly, freezing effectively reduces the amount of water in an emulsion by converting liquid water to a somewhat inert form that is unable to hydrate emulsifier molecules adsorbed to droplet surfaces, thus promoting droplet-droplet interactions. Thirdly, due to the formation of ice crystals, the ionic strength of liquid in nonfrozen areas around emulsion droplets increases and thus may block electrostatic repulsion between charged droplets. Fourth, when the emulsion freezes, ice crystals form that penetrate the oil droplets, destroying the interfacial film at the oil-water interface and thus facilitating emulsion coalescence after thawing. Fifth, the emulsifier may adsorb to ice crystal surfaces, thus promoting competition between oil droplet surfaces and ice crystal surfaces for the emulsifier. As an additional mechanism, physicochemical changes in the protein may occur during the freezing of the emulsion, thereby altering its functional properties [2,32,37]. Freeze-thaw stability of an emulsion is closely related to its interface and emulsifying properties, and emulsification is an important functional property of protein emulsions used as frozen food ingredients [13]. Mechanistically, an increase in structural flexibility and emulsification of a protein can speed up its adsorption to the oil-water interface, facilitating the production of fine and stable droplets [39]. For example, Tang et al. (2013) studied the relationship between the structural and functional properties of bovine serum protein and found that greater protein flexibility correlated with a greater ability to form a strong viscoelastic protein film at the emulsion interface as an indicator of enhanced emulsifying properties [40]. As another example, Kato et al. (1985) studied correlations between trypsin sensitivities of different proteins and protein flexibility and found significant correlations between foaming behavior, emulsifying properties and protein flexibility. Therefore, protein flexibility is thought to greatly influence emulsifying and foaming properties [41]. As yet another example, Poon et al. (2001) generated proteins with different flexibilities by breaking S-S and non-covalent bonds, then explored the relationship between protein flexibility and emulsification ability and noted that an increase in protein flexibility led to improved emulsification ability [42]. Furthermore, research by Zang et al. (2018) showed that an increase in protein flexibility improved emulsification and increased the ability of an emulsion to resist changes in the external environment (such as freezing and thawing) [32], with a similar conclusion reached by Wang et al. (2020) [1]. Mechanistically, an increase in protein flexibility can expose more hydrophobic groups, enabling protein molecules to be quickly adsorbed in stable orientations at the oil-water interface, where they form a viscoelastic interface film as an indicator of improved emulsion freeze-thaw stability. Finally, Cabezas et al. (2019) reported that the improvement of emulsion freeze-thaw stability is directly related to the combined enhancement of emulsifying and structural properties of proteins found at the oil-water interface [21].

#### 3.2.3. Effect of Freeze-Thaw Cycles on MPI Emulsion Microstructure

**Figure 3:**
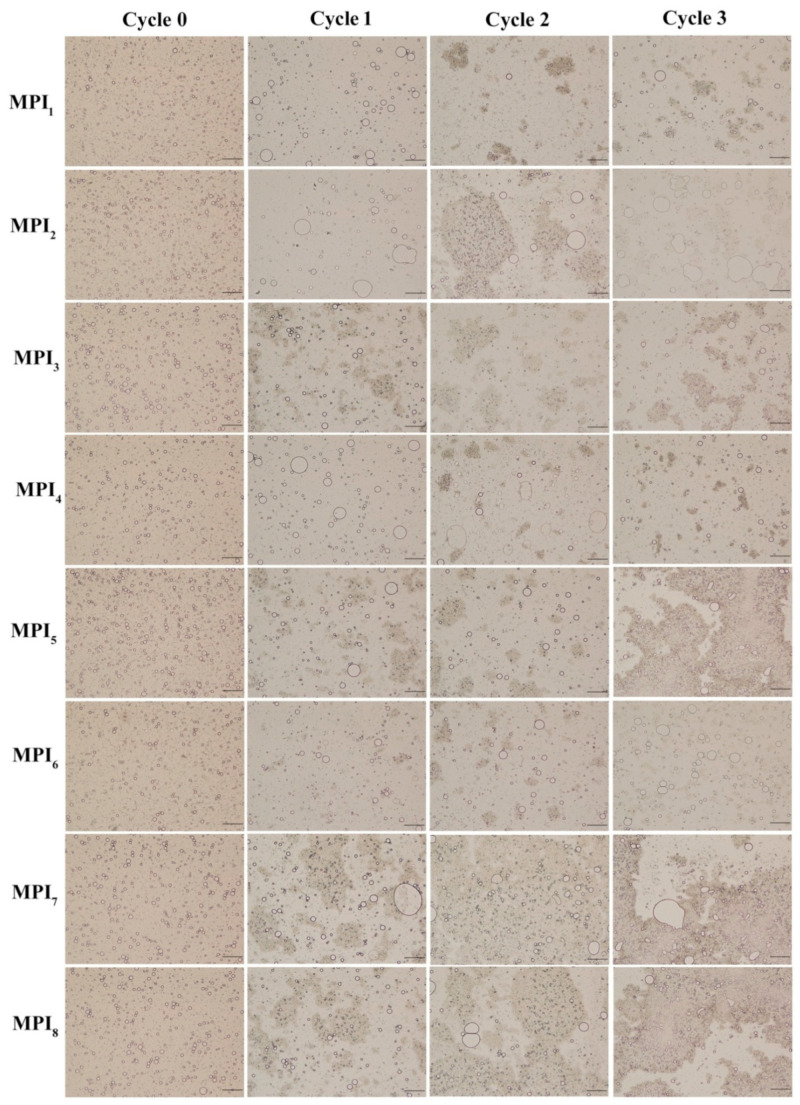
Optical micrographs of MPI emulsions before and after 0, 1, 2 and 3 freeze-thaw cycles (in order: MPI_1_–MPI_8_). Scale bar is 200 μm.

Micrographs are helpful for use in intuitively monitoring emulsion aggregation state. Figure 3 shows microstructures of eight protein-stabilised emulsions generated from proteins obtained from eight mung bean varieties before and after various numbers of freeze-thaw cycles. These images reveal substantial microscopic morphological differences among the emulsions before and after freeze-thawing. Specifically, droplets of all eight MPI emulsions (MPI_1_, MPI_2_, MPI_3_, MPI_4_, MPI_5_, MPI_6_, MPI_7_, MPI_8_) were uniformly distributed before freeze-thaw treatment and possessed similar microscopic morphologies. However, after multiple freeze-thaw cycle exposures, emulsion microstructures changed significantly. For example, irregularly shaped aggregates appeared in emulsions, while droplet distributions became uneven, an especially unfavourable freeze-thaw characteristic. Moreover, emulsion flocculation and coalescence increased with the increasing number of freeze-thaw cycles. Microscopic morphological results for the eight emulsions are summarised in Table 2 and are visualised in graphs depicted in Figure 2. Gu et al. (2007) found that after an emulsion stabilised by protein-based emulsification alone was subjected to freeze-thawing, emulsion particle size increased significantly, and droplet flocculation and coalescence occurred [43]. In other studies, including those conducted by Zhu (2020), Wang (2020) and Zang (2018), it was observed that a protein emulsion structure tended to collapse completely after freeze-thawing, as evidenced by microscopic detection of large clumps within the field of view and separation of oil and protein phases, as demonstrated in this study [1,32,44]. The underlying reason for these observations may be that the original protein, which was physically and chemically unmodified, may have had relatively poor solubility that rendered it unable to quickly adsorb to the oil-water interface to form a thick protective film [2]. In addition, freeze-thaw exposure may have induced low-temperature denaturation of the protein, causing the protein emulsifier in the emulsion to lose its emulsification ability [45]. In this case, when an emulsion freezes, the water in the system is redistributed as the volume and number of ice crystals increase, resulting in the piercing and disruption of the protein interface membrane and the formation of fat crystals between oil droplets that penetrate one another. After thawing, oil droplets aggregate, resulting in the production of irregular clumps [1].

### 3.3. Correlation Analysis

Due to scientific and technological developments in recent years, a variety of experimental methods now exist for use in evaluating protein structural quality and freeze-thaw stability of protein emulsions. However, correlation analyses of a large number of experimental indicators can often unnecessarily expand the data analysis workload. Therefore, a comparative analysis was employed here to detect significant correlations between the original protein structure, related functional properties and freeze-thaw stability of protein emulsions in order to clarify relationships among indicators to reduce the number of indicators needed for the final analysis. Importantly, analysis of emulsion freeze-thaw stability from perspectives of protein composition, subunit composition and emulsifying properties has far-reaching significance for the future development of applications for MPI resources, identification of special MPI characteristics and development of special mung bean varieties.

Table 3 lists Pearson correlation coefficients for protein structural indexes, emulsifying property characteristic indexes and freeze-thaw stability indexes for the MPI emulsions tested here. As shown in Table 3, the albumin content of MPI was negatively correlated with zeta potential (−0.745); the detection of a subunit band with a molecular weight of 26.9 kDa was positively correlated with particle size and oiling-off of the MPI emulsion after a freeze-thaw cycle (correlation coefficients were 0.794 and 0.769, respectively); flexibility value and emulsion stability value were negatively correlated with emulsion particle size, oiling-off, CI and zeta potential values after a freeze-thaw cycle (correlation coefficients of the four indicators with flexibility values were −0.820, −0.919, −0.769 and −0.823, respectively; correlation coefficients of the four indicators with emulsion stability values were −0.781, −0.771, −0.715 and −0.848, respectively). Moreover, the EAI was negatively correlated with the oiling-off value of the mung bean protein emulsion after freeze-thaw treatment (−0.720). Taken together, the abovementioned results revealed that MPI albumin content, the presence of a 26.9 kDa subunit band, flexibility value, emulsifying activity value and emulsifying stability value were key factors affecting the freeze-thaw stability of MPI emulsions. Thus, when used as an emulsifier, an MPI with certain characteristics (high albumin content, low content of the 26.9-kDa subunit, high EAI, high ESI and high flexibility value) can stabilize the emulsion and endow it with high freeze-thaw stability, making it suitable for use as an emulsifier in a freezing environment. Based on CI (Table 2 and Figure S1) and oiling-off values (Table 2) of MPI-stabilized emulsions of mung bean varieties MB1−8, it can be seen that the freeze-thaw stability of the MPI_4_-stabilized emulsion was superior to that of the other MPI-emulsions. Moreover, after three freeze-thaw cycles, MPI_4_ CI and oiling-off values were lowest (12.87% and 16.10%, respectively), while 26.9-kDa subunit band content, albumin content, protein flexibility value, EAI and ESI values for MPI_4_ were 12.89%, 310.3 mg/g of protein, 0.182, 8.598 m^2^/g and 28.05%, respectively.

## 4. Conclusions

The eight emulsions tested here, which were each stabilized by an MPI as the only emulsifier, all exhibited emulsion instability after one to three freeze-thaw cycles. However, due to mung bean variety-based differences in structural and emulsifying properties of MPIs, creaming index, oiling-off, particle size and zeta potential values of the eight emulsions significantly differed after freeze-thaw exposure. The emulsion stabilized by MPI_4_ possessed the lowest CI and oiling-off values after multiple freeze-thaw cycles, thus indicating its freeze-thaw stability was superior to that of the other MPI-stabilized emulsions. Compared with the unmodified SPI-stabilized emulsion that we previously studied, the MPI_4_-stabilized emulsion had lower CI and oiling-off values after multiple freeze-thaw cycles. Therefore, MPI could be used as an emulsifier to stabilize emulsified foods stored in frozen environments. Correlation analysis indicated that MPI, with low 26.9-kDa subunit content, high albumin content, emulsifying property and flexibility, its emulsion possessed high freeze-thaw stability and thus would be a suitable emulsifier when used in a frozen environment. Based on these results, future research studies should focus on how to modify the original MPI structure so as to improve the freeze-thaw stabilities of MPI-stabilized emulsions. In addition, protein structures and freeze-thaw stabilities of MPI and other plant proteins (e.g., SPI) should be compared in order to further reveal relationships between protein structural features and freeze-thaw stabilities. MPI possesses many functional properties that have not been well-utilised by the food industry. The research presented here provides a theoretical basis for improving mung bean applicability as an ingredient in frozen foods. These results should also promote the breeding and cultivation of special mung bean varieties for use in food production.

## Figures and Tables

**Table 1:** Structure and emulsification characteristic parameters of proteins from eight mung beans.

Parameter	Mung Bean Protein Samples							
	MPI_1_	MPI_2_	MPI_3_	MPI_4_	MPI_5_	MPI_6_	MPI_7_	MPI_8_
Albumin content (mg/g)	309.2 ± 3.79 ^a^	270.1 ± 5.00 ^b^	236.7 ± 4.37 ^c^	310.3 ± 5.38 ^a^	261.6 ± 2.40 ^b^	212.9 ± 9.42 ^d^	241.3 ± 6.98 ^c^	188.4 ± 2.03 ^e^
Globulin content (mg/g)	492.7 ± 5.06 ^a^	315.0 ± 3.33 ^e^	356.7 ± 1.07 ^c^	321.5 ± 2.54 ^d^	483.4 ± 5.60 ^b^	301.1 ± 0.09 ^f^	311.5 ± 0.10 ^e^	350.25 ± 3.47 ^c^
63.1 kDa (%)	24.87 ± 0.17 ^b^	24.93 ± 0.03 ^b^	23.81 ± 0.11 ^c^	25.20 ± 0.20 ^b^	23.57 ± 0.47 ^c^	23.72 ± 0.52 ^c^	22.55 ± 0.15 ^d^	26.18 ± 0.18 ^a^
52.9 kDa (%)	40.07 ± 0.07 ^d^	42.82 ± 0.32 ^b^	45.35 ± 0.25 ^a^	45.02 ± 0.02 ^a^	42.58 ± 0.18 ^b^	40.97 ± 0.20 ^c^	39.22 ± 0.22 ^e^	36.69 ± 0.19 ^f^
31.4 kDa (%)	13.59 ± 0.11 ^d^	13.02 ± 0.02 ^e^	13.06 ± 0.06 ^e^	13.18 ± 0.06 ^e^	13.28 ± 0.03 ^e^	13.94 ± 0.18 ^c^	14.53 ± 0.03 ^b^	15.31 ± 0.21 ^a^
26.9 kDa (%)	13.61 ± 0.11 ^c^	14.02 ± 0.02 ^b^	13.12 ± 0.12 ^d^	12.89 ± 0.19 ^d^	13.86 ± 0.16 ^bc^	14.04 ± 0.04 ^b^	15.09 ± 0.09 ^a^	13.91 ± 0.16 ^bc^
21.4 kDa (%)	7.86 ± 0.06 ^b^	5.21 ± 0.11 ^e^	4.67 ± 0.17 ^f^	3.70 ± 0.20 ^g^	6.71 ± 0.21 ^d^	7.34 ± 0.14 ^c^	8.61 ± 0.11 ^a^	7.91 ± 0.06 ^b^
α-helix (%)	17.92 ± 0.12 ^d^	20.10 ± 0.10 ^b^	19.33 ± 0.23 ^c^	20.42 ± 0.22 ^b^	21.72 ± 0.22 ^a^	17.92 ± 0.20 ^d^	19.48 ± 0.08 ^c^	17.76 ± 0.20 ^d^
β-sheet (%)	32.74 ± 0.08 ^a^	29.67 ± 0.10 ^c^	30.59 ± 0.05 ^b^	30.32 ± 0.03 ^b^	28.15 ± 0.27 ^d^	32.74 ± 0.28 ^a^	30.60 ± 0.12 ^b^	32.86 ± 0.27 ^a^
β-turn (%)	16.50 ± 0.25 ^b^	16.71 ± 0.21 ^ab^	16.58 ± 0.08 ^ab^	16.91 ± 0.12 ^a^	16.98 ± 0.08 ^a^	16.50 ± 0.10 ^b^	16.64 ± 0.04 ^ab^	16.35 ± 0.15 ^b^
Random coils (%)	32.83 ± 0.13 ^d^	33.51 ± 0.11 ^a^	33.42 ± 0.12 ^ab^	32.34 ± 0.04 ^e^	33.15 ± 0.15 ^bc^	32.83 ± 0.13 ^d^	33.28 ± 0.08 ^abc^	33.03 ± 0.03 ^cd^
Flexibility	0.161 ± 0.01 ^b^	0.149 ± 0.01 ^c^	0.145 ± 0.01 ^cd^	0.182 ± 0.01 ^a^	0.148 ± 0.01 ^c^	0.137 ± 0.01 ^d^	0.125 ± 0.01 ^e^	0.140 ± 0.01 ^cd^
EAI (m^2^/g)	8.439 ± 0.46 ^a^	7.687 ± 0.16 ^b^	7.569 ± 0.20 ^b^	8.598 ± 0.15 ^a^	7.564 ± 0.23 ^b^	6.735 ± 0.29 ^c^	7.293 ± 0.23 ^bc^	7.451 ± 0.24 ^bc^
ESI (%)	34.25 ± 0.92 ^a^	27.36 ± 0.99 ^b^	25.96 ± 0.48 ^b^	28.05 ± 1.04 ^b^	25.49 ± 1.25 ^bc^	22.94 ± 1.23 ^cd^	20.13 ± 1.09 ^d^	22.72 ± 1.15 ^cd^

Note: Notation of data within the same row with different letters indicates that the data are significant (*p* < 0.05).

**Table 2:** Freeze-thaw stability parameters of MPI emulsions.

Parameter	Mung Bean Protein Emulsion Samples							
	MPI_1_	MPI_2_	MPI_3_	MPI_4_	MPI_5_	MPI_6_	MPI_7_	MPI_8_
D_4,3_-0 (nm)	343.4 ± 10.40 ^f^	386.9 ± 14.90 ^de^	428.9 ± 8.90 ^abc^	376.5 ± 6.50 ^e^	406.5 ± 6.50 ^bcd^	451.7 ± 11.70 ^a^	432.3 ± 12.30 ^ab^	403.7 ± 3.70 ^cde^
D_4,3_-1 (nm)	453.8 ± 7.80 ^e^	605.8 ± 5.80 ^d^	707.2 ± 12.20 ^c^	460.0 ± 15.00 ^e^	750.8 ± 15.80 ^bc^	636.7 ± 16.70 ^d^	1156.0 ± 46.00 ^a^	769.4 ± 17.40 ^b^
D_4,3_-2 (nm)	676.7 ± 10.70 ^e^	640.8 ± 5.80 ^f^	1982.0 ± 14.00 ^a^	790.2 ± 12.20 ^d^	866.2 ± 10.20 ^c^	779.9 ± 4.90 ^d^	1196.0 ± 7.00 ^b^	877.2 ± 7.20 ^c^
D_4,3_-3 (nm)	722.9 ± 7.90 ^g^	1054.0 ± 19.00 ^d^	1997.0 ± 37.00 ^a^	836.8 ± 6.80 ^f^	945.7 ± 9.70 ^e^	960.6 ± 4.60 ^e^	1317.0 ± 7.00 ^b^	1227 ± 7.00 ^c^
Oiling off-1 (%)	16.21 ± 0.21 ^f^	18.14 ± 0.24 ^e^	21.03 ± 0.23 ^c^	14.12 ± 0.12 ^g^	21.38 ± 0.28 ^bc^	20.02 ± 0.02 ^d^	26.53 ± 0.33 ^a^	21.84 ± 0.24 ^b^
Oiling off-2 (%)	16.86 ± 0.09 ^g^	20.02 ± 0.02 ^f^	23.50 ± 0.10 ^c^	15.32 ± 0.15 ^h^	21.84 ± 0.16 ^d^	21.38 ± 0.09 ^e^	26.94 ± 0.12 ^a^	25.25 ± 0.06 ^b^
Oiling off-3 (%)	16.95 ± 0.07 ^f^	21.84 ± 0.09 ^e^	23.69 ± 0.10 ^c^	16.10 ± 0.08 ^g^	22.49 ± 0.08 ^d^	23.50 ± 0.09 ^c^	31.09 ± 0.09 ^a^	26.53 ± 0.05 ^b^
CI-1 (%)	15.00 ± 0.60 ^de^	21.67 ± 0.27 ^c^	29.52 ± 0.22 ^b^	10.00 ± 1.00 ^e^	30.95 ± 0.45 ^b^	20.58 ± 0.28 ^c^	57.14 ± 3.14 ^a^	53.94 ± 1.94 ^a^
CI-2 (%)	16.41 ± 0.16 ^f^	28.00 ± 0.50 ^e^	35.00 ± 0.80 ^c^	12.00 ± 1.00 ^g^	35.00 ± 0.80 ^c^	30.95 ± 0.20 ^d^	59.00 ± 1.00 ^a^	55.00 ± 1.50 ^b^
CI-3 (%)	20.20 ± 0.20 ^g^	29.52 ± 0.52 ^f^	38.10 ± 0.60 ^d^	12.87 ± 0.87 ^h^	43.37 ± 0.37 ^c^	34.76 ± 0.76 ^e^	59.64 ± 0.49 ^b^	64.11 ± 0.61 ^a^

Note: (1) 0–3 represents 0–3 freeze-thaw cycles; (2) Notation of data within the same row with different letters indicates that the data are significant (*p* < 0.05).

**Table 3:** Correlation coefficient.

	Albumin Content	Globulin Content	63.1 (kDa)	52.9 (kDa)	31.4 (kDa)	26.9 (kDa)	21.4 (kDa)	α-Helix	β-Sheet	β-Turn	Random Coils	Flexibility	EAI	ESI
D_4,3_	−0.539	−0.402	−0.607	−0.407	0.523	0.794 *	0.532	0.083	0.049	−0.099	0.515	−0.820 *	−0.585	−0.781 *
Oiling off	−0.682	−0.292	−0.591	−0.458	0.546	0.769 *	0.606	−0.031	0.157	−0.237	0.604	−0.919 **	−0.720 *	−0.771 *
CI	−0.691	−0.130	−0.210	−0.652	0.682	0.682	0.610	−0.146	0.064	−0.359	0.463	−0.769 *	−0.507	−0.715 *
Zeta potential	−0.745 *	−0.277	−0.469	−0.466	0.622	0.655	0.547	0.024	0.280	−0.168	0.440	−0.823 *	−0.698	−0.848 **

Note: * means significant, *p* < 0.05, ** means extremely significant, *p* < 0.01.

## Data Availability

The data presented in this study are available on request from the corresponding author.

## Supplementary Materials

The following supporting information can be downloaded at: https://www.mdpi.com/article/10.3390/foods11213343/s1, Figure S1: Photographs of MPI emulsions before and after 0, 1, 2 and 3 freeze-thaw cycles; 1, 2, 3 and 4 represent 0, 1, 2 and 3 freeze-thaw cycles, respectively.

## Abbreviations

MPI: mung bean protein isolate; SPI: soy protein isolate; MB: mung bean; FT-IR: fourier-transform infrared spectroscopy; CD: circular dichroism; EAI: emulsifying activity index; ESI: emulsifying stability index; CI: creaming index; MPIs: Mung bean protein isolates.
