# Supplementary material for: Study on Protein Structures of Eight Mung Bean Varieties and Freeze-Thaw Stability of Protein-Stabilized Emulsions
Source: Foods. 2022 Oct 24;11(21):3343. doi: 10.3390/foods11213343 (PMC9655322; doi:10.3390/foods11213343)
Supplement: Supplementary file 1 [file foods-11-03343-s001.zip › foods-1888026-supplementary.pdf]

Supplementary Material

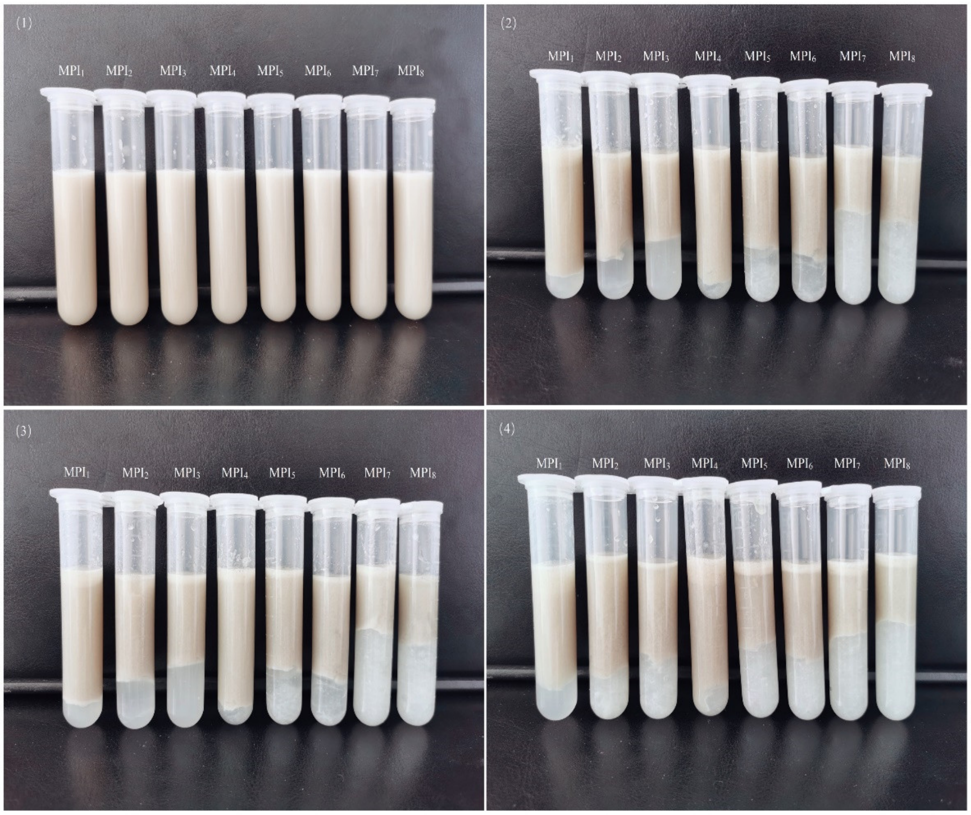

Figure S1. Photographs of MPI emulsions before and after 0, 1, 2 and 3 freeze-thaw cycles; 1, 2, 3 and 4 represent 0, 1, 2 and 3 freeze-thaw cycles, respectively.
